# Poly(ethylene glycol)-Based Hydrogel Microcarriers Alter Secretory Activity of Genetically Modified Mesenchymal Stromal Cells

**DOI:** 10.1021/acsbiomaterials.3c00954

**Published:** 2023-10-31

**Authors:** Gilad Doron, Levi B. Wood, Robert E. Guldberg, Johnna S. Temenoff

**Affiliations:** † Wallace H. Coulter Department of Biomedical Engineering, Georgia Tech and Emory University, 313 Ferst Dr. NW, Atlanta, Georgia 30332, United States; ‡ George W. Woodruff School of Mechanical Engineering, Georgia Institute of Technology, 801 Ferst Dr. NW, Atlanta, Georgia 30318, United States; § Parker H. Petit Institute for Bioengineering and Bioscience, Georgia Institute of Technology, 315 Ferst Dr. NW, Atlanta, Georgia 30332, United States; ∥ Knight Campus for Accelerating Scientific Impact, University of Oregon, 6231 University of Oregon, Eugene, Oregon 97403, United States

**Keywords:** mesenchymal stromal cell, poly(ethylene glycol) hydrogel, microcarrier, genetic modification, MAPK

## Abstract

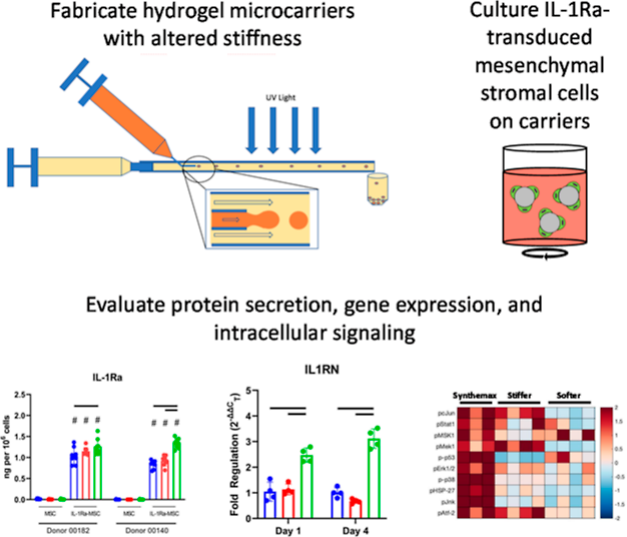

In order to scale up culture therapeutic cells, such as mesenchymal stromal cells (MSCs), culture in suspension bioreactors using microcarriers (μCs) is preferred. However, the impact of microcarrier type on the resulting MSC secretory activity has not been investigated. In this study, two poly(ethylene glycol) hydrogel formulations with different swelling ratios (named “stiffer” and “softer”) were fabricated as μC substrates to culture MSCs and MSCs genetically modified to express the interleukin-1 receptor antagonist (IL-1Ra-MSCs). Changes in cell number, secretory and angiogenic activity, and changes in MAPK signaling were evaluated when cultured on hydrogel μCs, as well as on tissue culture plastic-based Synthemax μCs. We demonstrated that culture on stiffer μCs increased secretion of IL-1Ra compared to culture on Synthemax μCs by IL-1Ra-MSCs by 1.2- to 1.6-fold, as well as their in vitro angiogenic activity, compared to culture on Synthemax μCs, while culture on both stiffer and softer μCs altered the secretion of several other factors compared to culture on Synthemax μCs. Changes in angiogenic activity corresponded with increased gene expression and secretion of hepatocyte growth factor by MSCs cultured on softer μCs by 2.5- to 6-fold compared to MSCs cultured on Synthemax μCs. Quantification of phosphoprotein signaling with the MAPK pathway revealed broad reduction of pathway activation by IL-1Ra-MSCs cultured on both stiffer and softer μCs compared to Synthemax, where phosphorylated c-Jun, ATF2, and MEK1 were reduced specifically on softer μCs. Overall, this study showed that μC surfaces can influence the secretory activity of genetically modified MSCs and identified associated changes in MAPK pathway signaling, which is a known central regulator of cytokine secretion.

## 1 Introduction

Mesenchymal stromal cells (MSCs) are highly active paracrine cells that have been used in clinical trials for treating various pathologies, including musculoskeletal, cardiovascular, neurodegenerative, and autoimmune diseases.^1^ Development of MSCs as clinical therapies has raised the need for technologies that can improve the efficiency of cell production. MSC therapies typically require doses of 10–100 million cells per patient, which necessitates cell culture processes that can economically manufacture batches of high-quality therapeutic cells at large scales.^2,3^ To reach these scale-up goals, various types of stirred-tank bioreactors have been explored for MSC culture,^3^ often using microcarriers (μCs).

μCs are solid spheres with diameters ranging from 100 to 300 μm that enable cell adhesion in suspension bioreactors. They present several different environmental cues to MSCs distinct from planar culture formats, including 3D architecture, substrate curvature, and convection-mediated transfer of nutrients in bioreactors.^4^ μCs have been shown to support MSC proliferation similarly to planar culture surfaces.^5^ However, the ability of μCs to modulate the MSC secretion of paracrine factors, a putative mechanism of action in vivo, is relatively unexplored. To date, MSCs cultured on dextran-based μCs have been found to increase secretion of interleukin-6 (IL-6) and interleukin-8 (IL-8) compared to those cultured on planar tissue culture plastic (TCP) surfaces coated with type I collagen.^5,6^ Specifically, the role of μC substrate stiffness on MSC proliferation and factor secretion has not yet been examined.^4^ Previous work by our laboratories and others has shown culturing MSCs on 2D surfaces with stiffness on the order of kPa reduces proliferation and amplifies secretion.^7−9^ Therefore, evaluating conditioning strategies, such as tuning μC stiffness to alter MSC proliferation and amplify secretory activity, may be useful for the development of highly therapeutic MSC therapies.

Genetic modification offers another strategy to alter the MSC secretome. Though the use of various types of viral and nonviral vectors differ in efficiency and safety,^10^ ex vivo genetic modifications before therapeutic delivery can induce expression and production of transgene-encoded therapeutic proteins. Doing so can confer a new mode of therapeutic function to MSCs or further augment an existing one while preserving their broad regenerative paracrine activity. This kind of ex vivo gene therapy for improving therapeutic protein secretion has shown success at treating a variety of diseases, depending on the therapeutic protein of interest. MSCs genetically modified to secrete interleukin-4 and -10 (IL-4 and IL-10) improved their ability to reduce the progression of osteoarthritis in preclinical studies,^11,12^ while MSCs modified to overexpress and secrete hepatocyte growth factor (HGF) improved their cell survival postimplantation and improved their ability to treat myocardial infarction in preclinical studies.^13^

Despite the benefits of genetically modified MSCs, current strategies for the genetic modification of MSCs result in low or transient transgene expression. Several promoters used to induce transgene expression are known to be repressed or silenced in MSCs and other stem cells.^14,15^ The cellular mechanisms responsible for this silencing in MSCs are still being explored, but studies using other cell types have identified epigenetic mediators,^14^ as well as intracellular signaling pathways like MAPK as regulating transgene expression.^16^

To overcome limited transgene expression in MSCs, many researchers have focused on optimizing gene delivery strategies, including the selection of viral delivery vehicles or the selection of optimal physiologic transduction methods.^15,17^ However, very few have evaluated strategies for improving transgene expression following gene delivery. Interestingly, the use of culture substrates significantly softer than those conventionally used has the potential for improving transgene expression in MSCs, as well as secretion of transgene-encoded proteins. In addition to amplifying MSC secretory activity,^7^ culture substrate stiffness has been shown to alter cellular mechanisms associated with transgene silencing, including the MAPK signaling pathways mentioned above.^18,19^ However, their capacity to do so using μC culture formats has not been explored.

Therefore, the objective of this study was to determine the effects of MSCs genetically modified to overexpress interleukin-1 receptor antagonist (IL-1Ra) and cultured on either TCP-based μCs (stiffness on the order of GPa^9^) or hydrogel μCs (stiffness on the order of kPa^20^) on secretion of IL-1Ra and other paracrine factors. IL-1Ra was chosen as a transgene target because it has shown preclinical success as a gene therapy for treating osteoarthritis.^21,22^ In particular, secretion of IL-1Ra and several other endogenously secreted factors were quantified. Resulting changes to functional activity were evaluated using an in vitro angiogenic assay. Because signaling within the MAPK pathway is a key regulator of cytokine expression, we also explored differences in the activation of this pathway between genetically modified MSCs cultured on different μCs. To our knowledge, this study is the first to evaluate the impact of substrate stiffness on MSC functionality using μC culture formats as well as the first to explore its potential to improve the secretory activity of genetically modified MSCs following gene delivery.

## 2 Materials and Methods

### 2.1 Material Synthesis and Functionalization

Poly(ethylene glycol) diacrylate (PEG-DA, 3.4 kDa, Sigma-Aldrich) was synthesized according to previously published methods.^23,24^ Integrin-engaging peptide RGD (GRGDS, Bachem) was conjugated to 3.4 kDa acrylate-PEG-SVA (LaysanBio) according to previous protocols to form Acryl-PEG-RGD.^25,26^ Greater detail can be found in the Supporting Information Methods.

### 2.2 μC Fabrication

**Figure 1:**
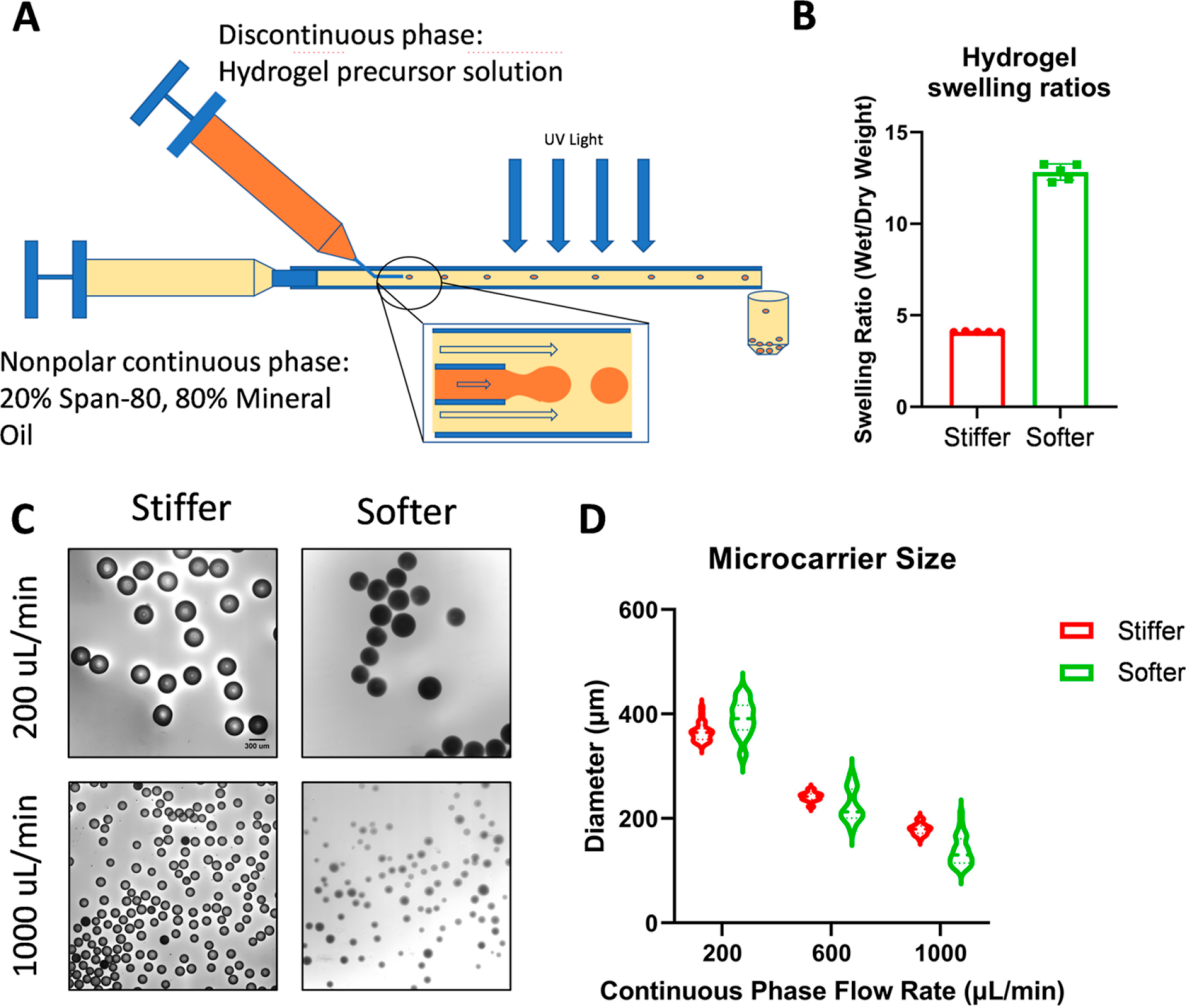
Fabrication of homogeneously sized hydrogel μCs of different compositions. (A) Schematic of fluidic device used to fabricate hydrogel substrates as μCs (schematic created by Elda Treviño). (B) Swelling ratios of hydrogels used to fabricate μCs of different stiffnesses. Swelling ratios of 20 wt % 575 Da PEG-DA hydrogels and 10 wt % 3.4 kDa PEG-DA hydrogels were not significantly different from those previously determined to have compressive moduli of 100 and 30 kPa, respectively (*p* > 0.05, one-way ANOVA). (C) Representative images of stiffer and softer μCs fabricated using different continuous phase flow rates. μCs were stained with trypan blue for imaging (scale bar = 300 μm). (D) Resulting μC diameters using different continuous phase flow rates (mean ± SD, *n* = 30).

Formulations of hydrogels were chosen that exhibit swelling ratios similar to those previously determined to have stiffnesses of 30 and 100 kPa,^7^ which will henceforth be referred to as “softer” and “stiffer” formulations, respectively. “Softer” μCs consisted of 10 wt % 3.4 kDa PEG-DA and 1 mM Acryl-PEG-RGD, and “stiffer” μCs consisted of 12.5 or 20 wt % 575 Da PEG-DA (Sigma), depending on the lot, and 1 mM Acryl-PEG-RGD. Acryl-PEG-RGD was used to enable cell binding to PEG μCs. A fluidic device designed previously in the laboratory was used to fabricate homogeneously sized hydrogel μCs (Figure 1A).^27^ Briefly, to construct the device, a 30-gauge needle (BD) was bent at a 45° angle and pierced through miniature ethylene-vinyl acetate tubing (0.04 in. inner tube diameter, McMaster-Carr). The needle was stabilized in a 1.7 mL microcentrifuge tube cap (VWR) using epoxy resin (Sigma) to prevent leaking. The tubing was coiled under a Blak-Ray long wave ultraviolet (UV) lamp (Model B, UVP) for 60 s of UV exposure. To operate the device, a continuous phase of 20% span-80 (Sigma) and 80% mineral oil (VWR) was pumped through the device at speeds ranging from 200 to 1000 μL/min. Hydrogel μCs were formed by pumping the discontinuous phase of hydrogel precursor solution mixed with photoinitiator lithium phenyl(2,4,6-trimethylbenzoyl)phosphinate (L0290, TCI) through the device at 5 μL/min. The resulting hydrogel μCs were collected and washed twice with light mineral oil (VWR), once with 0.26% Pluronic F-127 (Sigma) in diH_2_O, and once with diH_2_O. μCs were then sterilized in 70% ethanol (Sigma) for 30 min and were washed twice in phosphate-buffered saline (PBS, Gibco). μCs were stored at 4 °C until their use and were used within a week of fabrication. μC size and concentration were quantified by staining with trypan blue (Gibco) and imaging them using a Nikon TE3000 fluorescence microscope at 4× magnification. Diameters were quantified using ImageJ software (NIH), which, assuming μCs were spherical, were used to calculate the average μC surface area.

### 2.3 Genetic Modification of MSCs

Bone marrow-derived MSCs from two donors (RoosterBio, lots 00182 and 00140) were separately cultured for 4 days in DMEM-LG (Gibco) supplemented in 10% fetal bovine serum (FBS, Atlanta Biologicals) and 1% antibiotic/antimycotic solution (Gibco). MSCs were then trypsinized and replated at 20,000 cells/cm^2^ and were allowed to adhere overnight. Cells were then incubated in culture media supplemented with AAV particles encoding the human gene IL1RN-IRES-EGFP (GeneCopoeia, AA02-A0332-AV08) at multiplicities of infection (MOI) of either 10^4^ or 10^5^ for 48 h. Unmodified MSCs were incubated with no AAV particles. Following AAV transduction, unmodified and genetically modified MSCs (IL-1Ra-MSCs) were lifted using TrypLE Express (Gibco) and used for planar or μC culture. Successful genetic modification was confirmed by evaluating EGFP expression using the Zeiss LSM 700 confocal microscope (Zeiss). Secretion of IL-1Ra by IL-1Ra-MSCs was confirmed by seeding MSCs onto 6-well TCP plates and collecting the resulting conditioned media (CM) after 4 days. IL-1Ra concentration in the CM was evaluated using the Human IL-1ra/IL-1F3 Quantikine ELISA kit (R&D systems).

### 2.4 μC Culture

TCP-based Synthemax II μCs (Synthemax, Corning) and stiffer and softer hydrogel μCs were incubated in MSC culture media specified previously for at least 30 min before the addition of cells. Following AAV transduction, unmodified and/or genetically modified MSCs were added to μC suspensions in 12-well Nunclon Sphera ultralow binding plates (ThermoFisher). Cells on μCs were cultured in 1 mL volume per well, with μC densities of 10 cm^2^ surface area/mL. Cells were seeded at 5000 cells/cm^2^ under static conditions for 24 h. Afterward, cells were agitated using an orbital shaker (Benchmark Scientific) at 97 rpm (0.1 RCF). Cells on μCs and CM were collected after 4 days of culture. All culture procedures using IL-1Ra-MSCs were performed using MSC culture media specified previously.

### 2.5 Quantification of MSC Number

To assess changes in cell number over the course of μC culture, MSCs, and IL-1Ra-MSCs from each donor cultured on different μCs were transferred to 1.7 mL microcentrifuge tubes and washed twice with PBS. Cells on μCs were incubated in TrypLE Express for 5 min before adding MSC culture media to quench the reaction. Cells and μCs were then washed with PBS and lysed by three freeze–thaw cycles. Cell number was quantified using a Quant-iT PicoGreen DNA assay kit (Invitrogen). Fold change in cell number was calculated by comparing the number of cells collected after 4 days in culture versus the initial number of cells seeded.

### 2.6 Secretome Characterization

CM from MSCs and IL-1Ra-MSCs on different μCs were collected after 4 days in culture. Media was centrifuged at 400*g* to remove any potential cell debris before freezing the supernatant at −80 °C in aliquots until use. CM was evaluated using a custom Luminex assay for 24 human cytokines, chemokines, and growth factors and a custom ProCartaPlex assay for HGF and SDF-1a (ThermoFisher). All analytes for secretome characterization are listed in Table S1. Data were read using the MAGPIX system (Luminex) and analyzed using Milliplex Analyst Software (EMD Millipore). Background levels of these factors from media were subtracted from final values, and production of all factors was normalized by the number of cells in the sample after 4 days in culture. Principal component analysis (PCA) was performed separately on each MSC donor to identify the factors contributing to the variance within the secretome contributed by genetic modification and μC formulation. Factors that were secreted below the limits of detection were excluded from PCA.

### 2.7 In Vitro Angiogenesis Assay

Human vascular endothelial cells (HUVECs, Lifeline Cell Technology) were culture expanded on 0.1% gelatin-coated flasks in Endothelial Media (R&D systems) for 1 passage prior to seeding into the angiogenesis assay. Lactate dehydrogenase-elevating virus (LDEV)-free Matrigel matrix (Corning) was plated in microwell slides (ibidi) and allowed to gel for 1 h at 37 °C. HUVECs were harvested with TrypLE Express (Gibco) and centrifuged. After removing the supernatant, cells were resuspended in MSC-CM (20,000 cells/30 μL) and plated over the Matrigel at 15 μL per microwell in duplicate and cells were maintained in a humidified chamber at 37 °C for 8 h. Each microwell was imaged at 4× magnification using a Nikon TE3000 fluorescence microscope and analyzed using ImageJ Angiogenesis Analyzer.^28^

### 2.8 Gene Expression of Genetically Modified MSCs

Gene expression of IL-1Ra-MSCs cultured on different μCs was performed to determine whether transgenes and endogenous genes whose secretion is increased on softer μCs corresponded to their transcriptional upregulation. Expression of IL-1Ra and two representative endogenous genes upregulated by softer μCs (HGF and IL-10) was evaluated using qRT-PCR. RNA from IL-1Ra-MSCs from each donor cultured on different μCs was isolated 1 and 4 days after seeding onto μCs. Cells on μCs were transferred to Falcon centrifuge tubes (VWR) and trypsinized. Cells were then passed through a 100 μm cell strainer (Fisher Scientific) to isolate cells. RNA from cells was then isolated using the RNeasy Micro kit (Qiagen) as specified by the manufacturer. RNA was stored at −80 °C until reverse transcription. 50 ng RNA was reverse transcribed using the High-Capacity cDNA Reverse Transcription Kit (ThermoFisher) as specified by the manufacturer. Primers for human IL1RN, HGF, IL-10, B2M, and GAPDH were purchased from a commercial vendor (Bio-Rad, Assays in Table S2). cDNA, primers, and PowerUp SYBR green master mix (Applied Biosystems) were reacted in 20 μL volumes using the StepOnePlus Real Time PCR system (ThermoFisher) as specified by the manufacturer. PCR results for IL1RN, HGF, and IL-10 were normalized to the geometric mean of housekeeping genes GAPDH and B2M as done previously.^26^ Gene expression was reported as fold regulation (2^–ΔΔ*CT*^).

### 2.9 MAPK Signaling of Genetically Modified MSCs

To assess changes in intracellular signaling by IL-1Ra-MSCs cultured on different μCs, we characterized cells for the presence of 10 individual MAPK phosphoproteins. Cells were cultured for ∼24 h prior to serum starvation to evaluate signaling in cells that were fully adhered to μCs. The cell lysate was collected using methods adapted from previous studies.^29^ IL-1Ra-MSCs from both donors were seeded onto μCs and incubated under static conditions for 24 h. Cells were then serum starved by removing 0.5 mL of the culture volume (50% of culture volume) and replacing it with 1 mL of DMEM-LG supplemented with 1× antibiotic antimycotic solution. After 30 min, 1 mL of culture volume (66.6% of culture volume) was removed and replaced with 0.5 mL media containing 16.6% FBS to return to yield an FBS fraction of 10%. μCs were then subjected to agitation as per the standard culture protocol (see Section 2.4).

Cells were lysed at 15, 60, and 120 min following the start of agitation. For lysis, cells on μCs were transferred to microcentrifuge tubes on ice, where they were washed twice with ice-cold PBS. Cells on μCs were then lysed in a Bio-Plex Cell Lysis kit (Bio-Rad) supplemented with Roche cOmplete Mini Protease Inhibitor Cocktail (1 tablet per 5 mL buffer) and 2 mM phenylmethylsulfonyl fluoride (PMSF, ThermoFisher) and placed on a tube rotator for 15 min at 4 °C and were placed at 4 °C at 16,600*g* for 10 min. The supernatant was aliquoted and stored at −80 °C until use. The total protein content of each sample was measured using a Pierce bicinchoninic acid (BCA) assay (ThermoFisher) with a Synergy H4Microplate reader (BioTek). MAPK pathway phospho-signaling proteins were quantified using the MAPK/SAPK signaling 10-plex kit (48-660MAG, EMD Millipore) and evaluated using the MAGPIX system. Median fluorescence intensity (MFI) values for each analyte were normalized to the protein content from each cell lysate sample.

### 2.10 Statistical Analysis

Sample sizes and replicates are stated in figure legends. Error bars represent the means ± standard deviation unless otherwise noted. Fold changes in cell number between MSCs and IL-1Ra-MSCs cultured on different μCs were analyzed using a two-way ANOVA with Tukey *posthoc* comparisons. Differences in the secretion of individual immunomodulatory factors were assessed using a two-way ANOVA with Tukey *posthoc* comparisons. Changes in angiogenic activity, gene expression, and MAPK phosphoprotein content between different μCs were assessed using a two-way ANOVA with Tukey *posthoc* comparisons. Analyses were performed using Prism software. PCA was used to discriminate secretory profiles of MSCs cultured on gel substrates, which were performed using SIMCA software (Umetrics). PCA was performed separately for each cell donor, with the respective *R*^2^ and *Q*^2^ values reported in the figure legends. Identification of candidate MAPK phosphoproteins discriminating between μC types was performed using a two-way ANOVA with Tukey *posthoc* univariate comparisons of individual MAPK proteins.

## 3 Results

### 3.1 Hydrogel μC Fabrication

Hydrogel μCs were fabricated using a novel fluidic device (Figure 1A).^27^ Hydrogels used to fabricate μCs exhibited swelling ratios similar to those of hydrogels previously characterized to have stiffnesses of 100 kPa (stiffer) and 30 kPa (softer) (Figure 1B). To determine the device operating parameters for fabricating μCs, hydrogel μCs of both stiffnesses were fabricated using nonpolar continuous flow rates of either 200, 600, or 1000 μL/min. Increasing the continuous phase flow rate was found to decrease the resulting diameters of both stiffer and softer hydrogel μCs (Figure 1C,D). At 200 μL/min, fabrication yielded stiffer and softer μCs with diameters of 365.2 ± 17.3 and 392.4 ± 33.7 μm, respectively (Figure 1C,D). At 600 μL/min, fabrication yielded stiffer and softer μCs with diameters of 241.9 ± 8.5 and 229.2 ± 36.4 μm, respectively (Figure 1D). At 1000 μL/min, fabrication yielded stiffer and softer μCs with diameters of 179.7 ± 10.2 and 138.0 ± 28.2 μm, respectively (Figure 1C,D). For all subsequent studies, continuous phase flow rates of 800 μL/min were used to fabricate μCs for cell culture, which yielded stiffer and softer μC diameters of 161.3 ± 11.6 and 196.0 ± 10.6 μm, respectively.

### 3.2 Genetic Modification of MSCs

In preliminary studies to generate IL-1Ra-MSCs, MSCs from a single cell donor (Donor 00140) were transduced at two different MOIs with AAV particles delivering genetic payload encoding for human IL-1Ra and EGFP. Confocal fluorescent microscopy revealed EGFP expression 4 days after ending viral transduction of MSCs at either 10^4^ or 10^5^ MOI (Figure S1A), confirming successful viral transduction and transgene expression. Evaluation of the IL-1Ra concentration in MSC-CM using a single-analyte ELISA demonstrated significant increases in IL-1Ra secretion by MSCs within 2 days following viral transduction at either MOI (Figure S1B). Greater MOIs were found to increase IL-1Ra secretion where over the course of 4 days MSCs transduced at 10^5^ MOI secreted 6.5 ± 1.5 ng/10^6^ cells/day and MSCs transduced at 10^4^ MOI secreted 3.2 ± 0.8 ng/10^6^ cells/day (Figure S1B). In all, AAV transduction successfully induced IL-1Ra secretion in MSCs. Successful induction of IL-1Ra secretion by MSCs was confirmed in another donor (Donor 00182) when using 10^5^ MOI (data not shown). All subsequent transductions used a 10^5^ MOI to maximize IL-1Ra secretion by MSCs.

### 3.3 IL-1Ra-MSC Number on Different μCs

**Figure 2:**
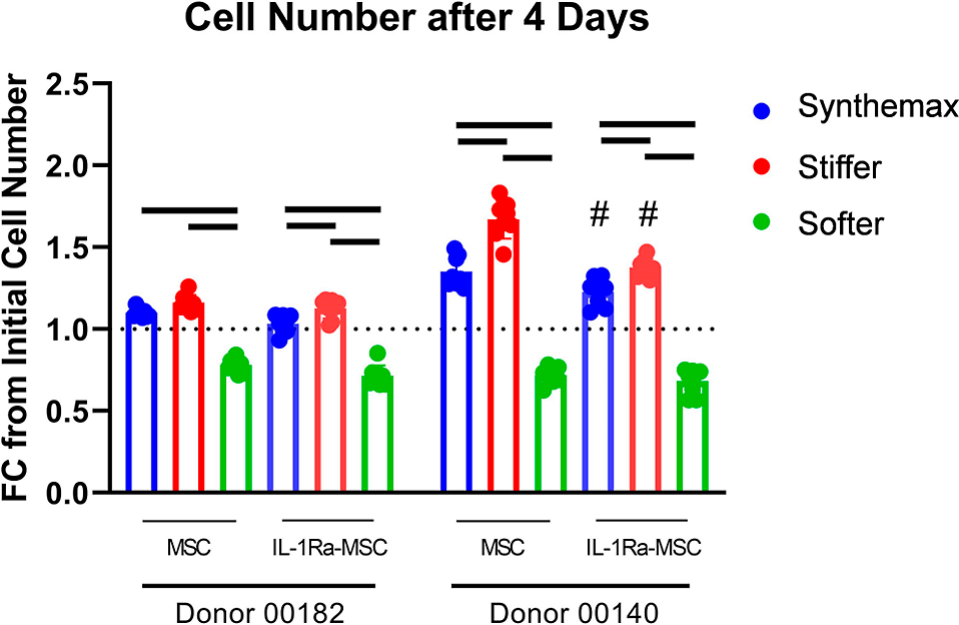
μC stiffness influences MSC and IL-1Ra-MSC number. Fold change (FC) in cell number at 4 days compared to initial cell number seeded on μCs. Dotted line indicates the number of cells seeded (mean ± SD, #*p* < 0.05 compared with MSCs on the same μC type, bars *p* < 0.05 between indicated groups; *n* = 8 per group).

Live cell imaging showed that MSCs and IL-1Ra-MSCs adhered to all of the μC types (Figure S2). MSCs and IL-1Ra-MSCs cultured on softer μCs yielded lower cell numbers compared with Synthemax or stiffer μCs (Figure 2). This effect of softer μCs was observed in both MSC donors. Synthemax and stiffer μCs supported limited MSC and IL-1Ra-MSC proliferation, where 1.0- to 1.7-fold increases in cell number were observed after 4 days of culture (Figure 2). IL-1Ra-MSCs cultured on stiffer μCs showed increased cell numbers for both donors compared with those cultured on Synthemax μCs (Figure 2). However, unmodified MSCs cultured on stiffer μCs yielded increased cell numbers only compared to those cultured on Synthemax μCs for Donor 0140 only (Figure 2). IL-1Ra-MSCs showed less of an increase in cell number than nonmodified MSCs on Synthemax and stiffer μCs in one cell donor, but this was not observed in the other donor (Figure 2).

### 3.4 MSC and IL-1Ra-MSC Secretory Activity Altered by Stiffer and Softer μCs

**Figure 3:**
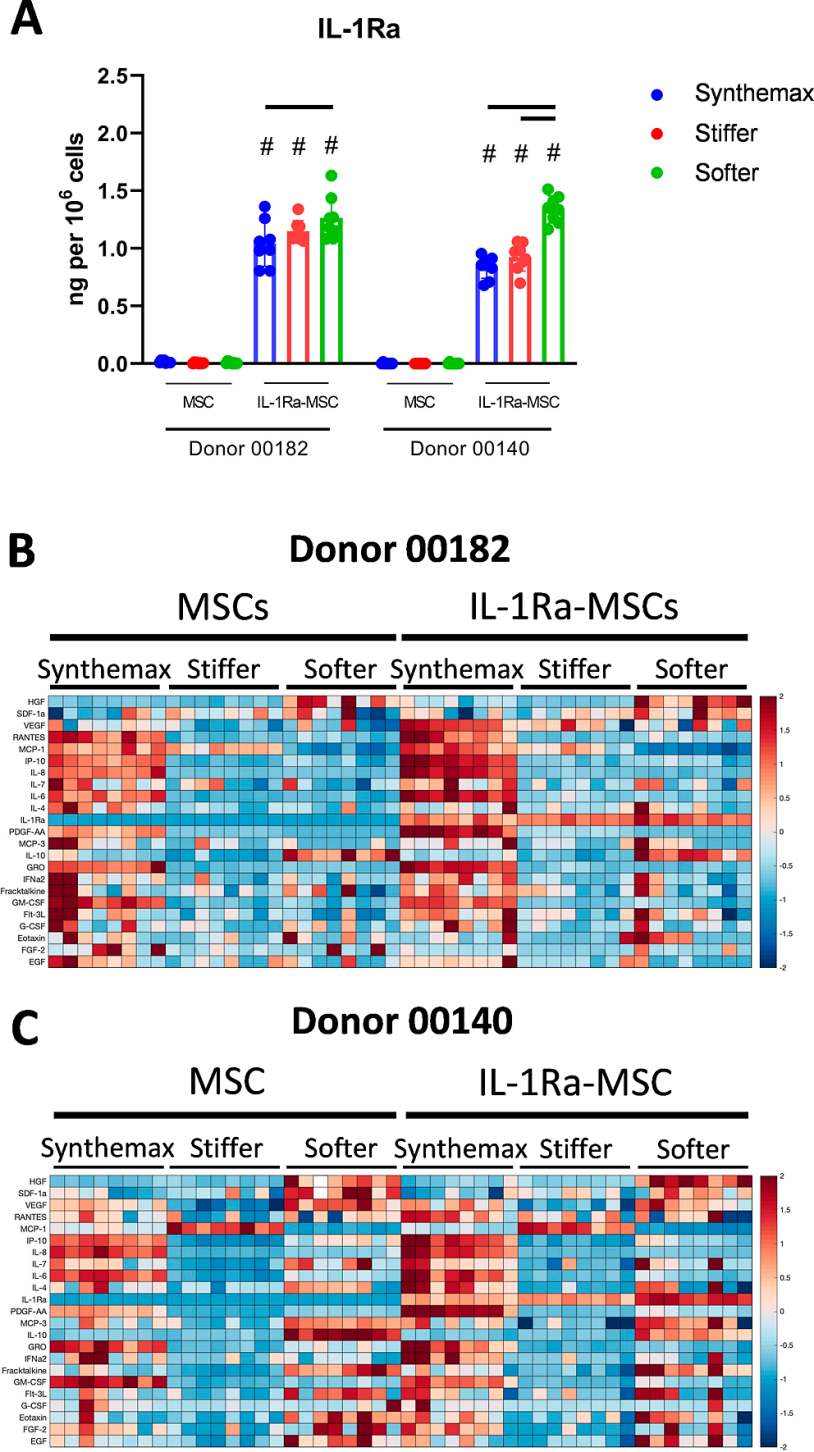
Softer μCs enhance the secretion of IL-1Ra by IL-1Ra-MSCs versus Synthemax μCs and alter the secretion of multiple additional immunomodulatory cytokines. (A) Softer μCs increase secretion of IL-1Ra by IL-1Ra-MSCs vs Synthemax μCs in two different MSC donors, Bars *p* < 0.05, #*p* < 0.05 vs MSCs on same μCs (two-way ANOVA, Tukey *posthoc*). (B,C) Relative abundance of additional secreted factors cultured on different μCs by MSCs of each donor, respectively, colors based on z-score.

Overall, both stiffer and softer μCs yielded distinct secretory profiles of both MSCs and IL-1Ra MSCs versus Synthemax μCs (Figure 3). IL-1Ra-MSCs cultured on softer μCs increased secretion of IL-1Ra compared to culture on Synthemax μCs in both donors but only increased secretion compared to culture on stiffer μCs in one cell donor (Figure 3A).

**Figure 4:**
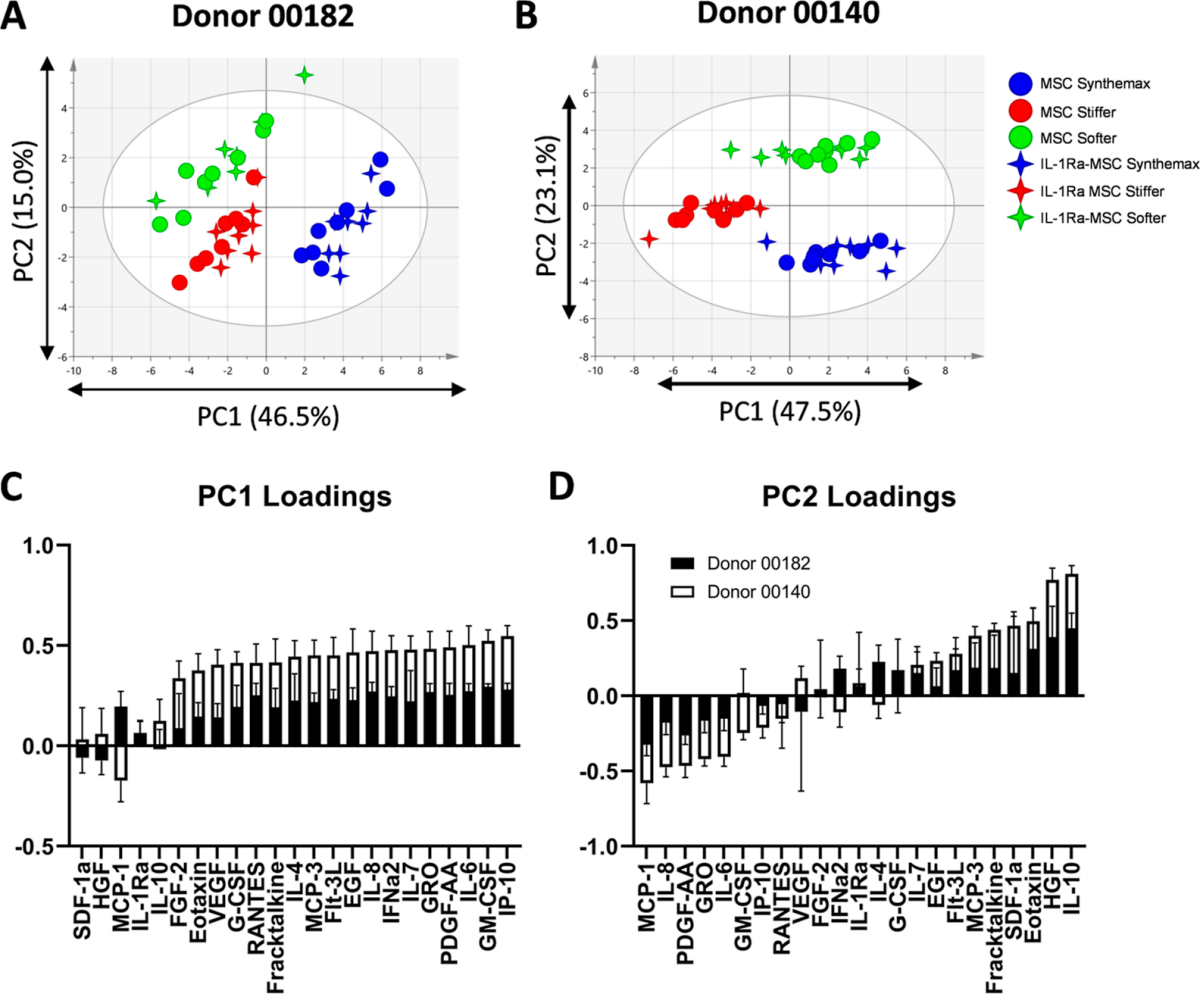
Synthemax, stiffer, and softer μCs yield distinct secretory profiles for both MSCs and IL-1Ra-MSCs. (A,B) PCA of MSC and IL-1Ra-MSC secretome on different μCs for each MSC donor shows separation between μCs along PC1 and PC2 (*n* = 8 per group). *R*^2^ = 0.614, 0.706 and *Q*^2^ = 0.485, 0.629 for donors 00182 and 00140, respectively. Secretory factor loading along (C) PC1 and (D) PC2 for both donors. Loadings for PCA performed separately for each donor are superimposed on each other.

Multivariate analysis with PCA performed separately for each donor showed distinct separation of MSC and IL-1Ra-MSC μCs based on the μC type (Figure 4A,B). Secretomes of MSCs showed no separation between those from IL-1Ra-MSCs cultured on the same μC type (Figure 4A,B). For both donors, MSCs and IL-1Ra-MSCs cultured on Synthemax μCs separated from those cultured on stiffer μCs along PC1, accounting for 46.5–47.5% of the variance in the data sets (Figure 4A,B). This separation was characterized by increased secretion of several cytokines by cells cultured on Synthemax μCs compared with cells cultured on hydrogel μCs, including IP-10, GM-CSF, IL-6, and PDGF-AA (Figure 4C). For both donors, cells cultured on softer μCs separated from Synthemax and stiffer μCs along PC2, accounting for 15.0–23.1% of the variance within the data set (Figure 4A,B). This separation was characterized by increased secretion of IL-10 and HGF and decreased secretion of MCP-1 and IL-8 on softer μCs (Figure 4D).

Analysis of individual analytes confirmed many of the distinctions in secretome observed between cells cultured on different μCs from PCA. MSCs and IL-1Ra-MSCs cultured on Synthemax μCs increased secretion of IP-10, PDGF-AA, GM-CSF, GRO, IL-6, and IL-8 compared to stiffer and softer μCs in both cell donors (Figure S3). MSCs and IL-1Ra-MSCs cultured on softer μCs showed increased secretion of HGF and IL-10 and decreased secretion of MCP-1 compared to Synthemax and stiffer μCs in both cell donors (Figure S3). Cells cultured on softer μCs also increased secretion of Fractalkine and SDF-1α in comparison to Synthemax and stiffer μCs, but only in one donor (Figure S3). MSCs and IL-1Ra-MSCs cultured on stiffer μCs demonstrated decreased secretion of VEGF and increased secretion of MCP-1 compared to that when cultured on Synthemax and softer μCs but only in one cell donor (Figure S3).

### 3.5 Softer μCs Increase MSC and IL-1Ra-MSC Angiogenic Activity

**Figure 5:**
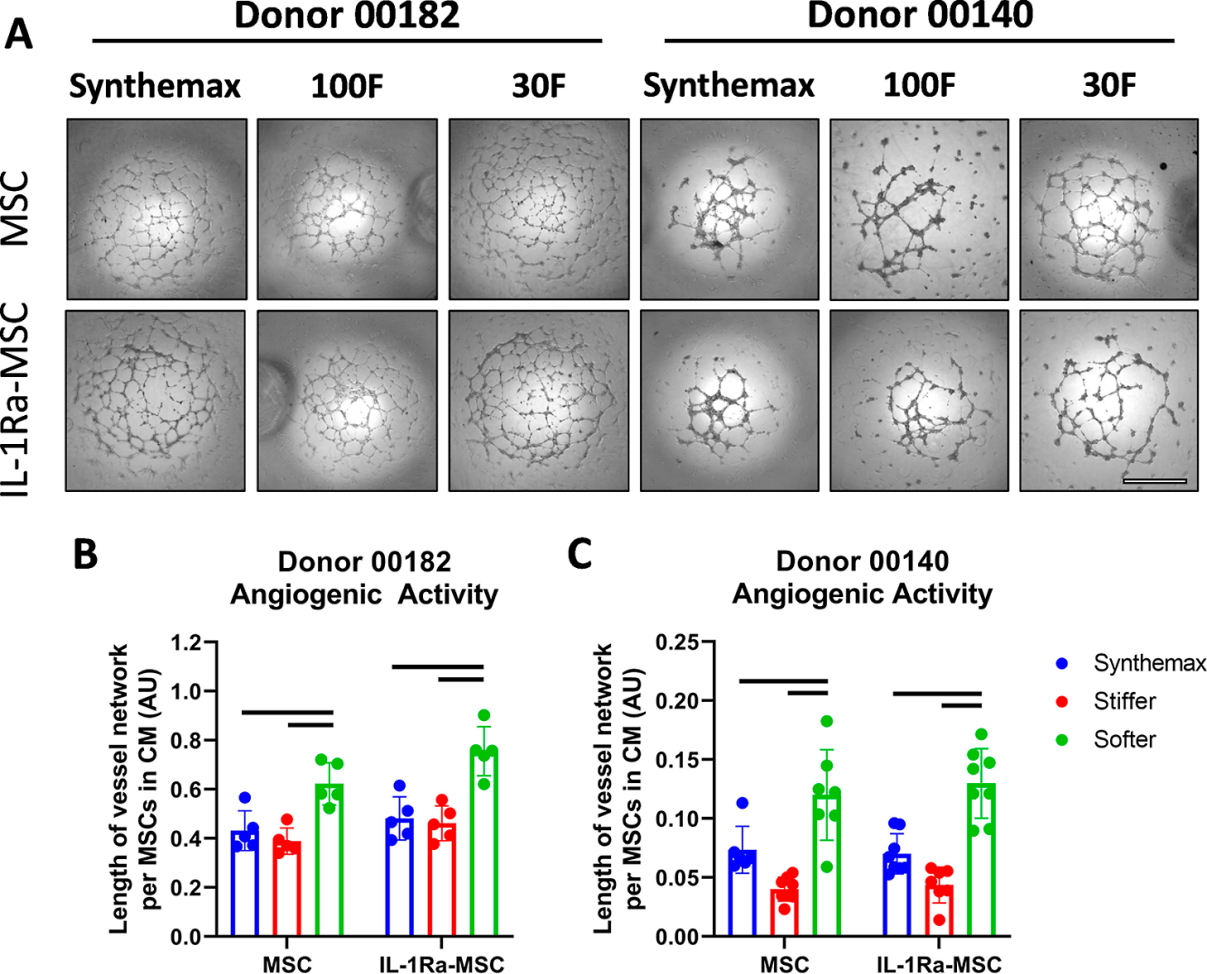
Softer μCs increase in vitro paracrine angiogenic activity of MSCs and IL-1Ra-MSCs. (A) Representative images of the HUVEC network formation on Matrigel with MSC and IL-1Ra-MSC-CM cultured on different μCs using two MSC donors (scale bar = 1000 μm). (B,C) Quantification of HUVEC network length in MSC and IL-1Ra-MSC-CM on different μCs using two MSC donors (*n* = 4–5, bars *p* < 0.05, two-way ANOVA, Tukey *posthoc*).

CM from cells cultured on softer μCs enhanced the HUVEC network formation compared with CM from cells cultured on Synthemax or stiffer μCs (Figure 5). No difference in angiogenic activity was observed between MSCs and IL-1Ra-MSCs cultured on the same type of μCs.

### 3.6 Softer μCs Increase Gene Expression of IL1RN and HGF

**Figure 6:**
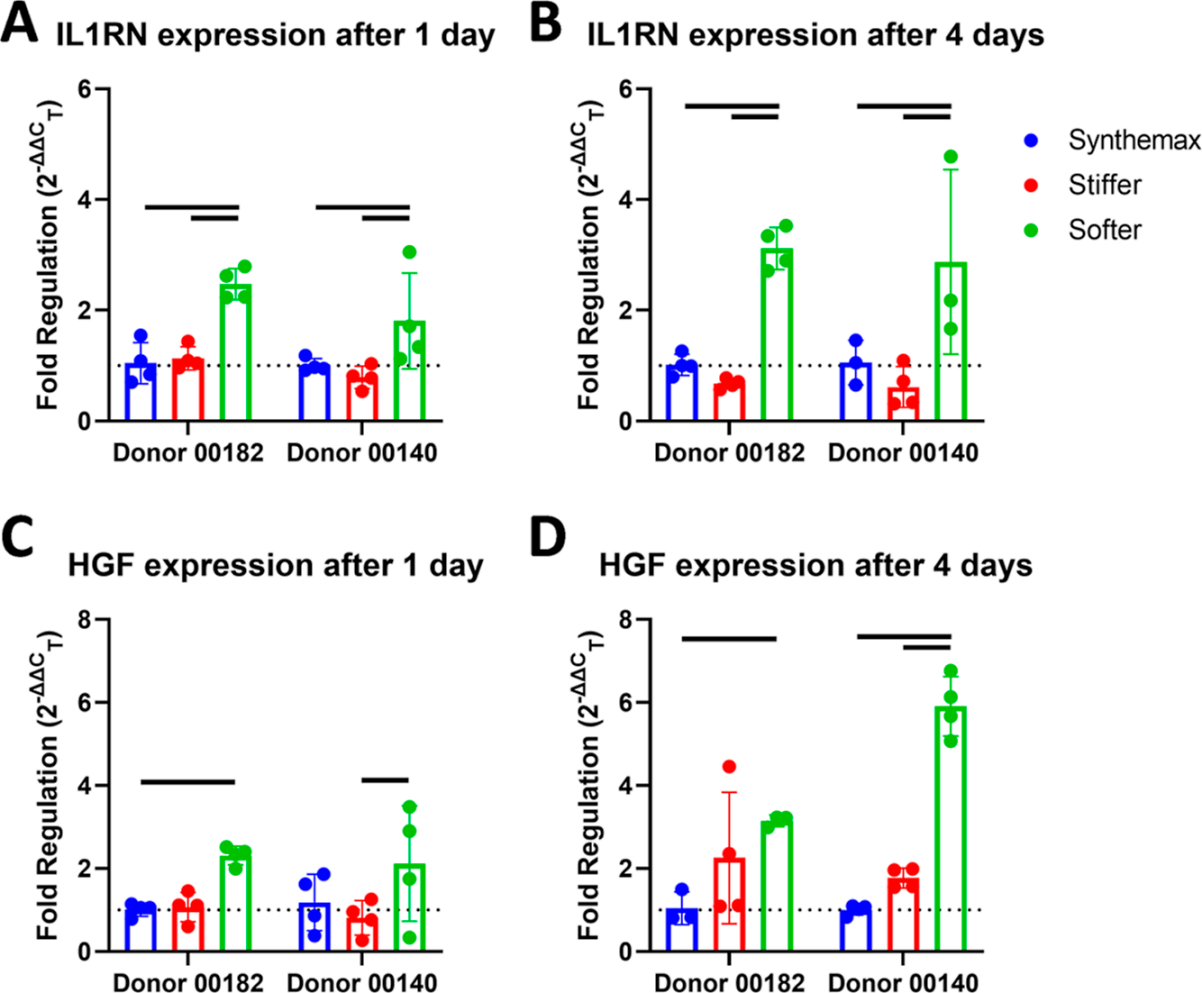
Softer μCs increase IL-1Ra and HGF expression by IL-1Ra-MSCs. (A) IL-1Ra (IL1RN) expression by IL-1Ra-MSCs from two donors cultured on different μCs 1 day after seeding, before the start of agitation and (B) 4 days after seeding. (C) HGF expression by IL-1Ra-MSCs from two donors cultured on different μCs 1 day after seeding, before the start of agitation, and (D) 4 days after seeding. Expression reported as fold regulation normalized to the geometric mean to two housekeeping genes. Reference gene expression values were the average of each donor cultured on Synthemax μCs at each time point. Bars *p* < 0.05 (*n* = 3–4, two-way ANOVA, Tukey *posthoc*).

While IL1RN and HGF gene expression was reliably detected in IL-1Ra-MSCs, IL-10 expression was not and was thus excluded from further transcript analysis. Culture on softer μCs resulted in increased expression of IL1RN compared to culture on Synthemax or stiffer μCs both 1 and 4 days after seeding in both cell donors (Figure 6A,B). For HGF, 1 day after seeding, cells cultured on softer μCs increased HGF expression versus either Synthemax or stiffer μCs in only one donor (Figure 6C). However, cells cultured on softer μCs did increase HGF expression versus Synthemax μCs in both donors 4 days after seeding (Figure 6D).

### 3.7 Stiffer and Softer μCs Both Alter MAPK Signaling in IL-1Ra-MSCs

**Figure 7:**
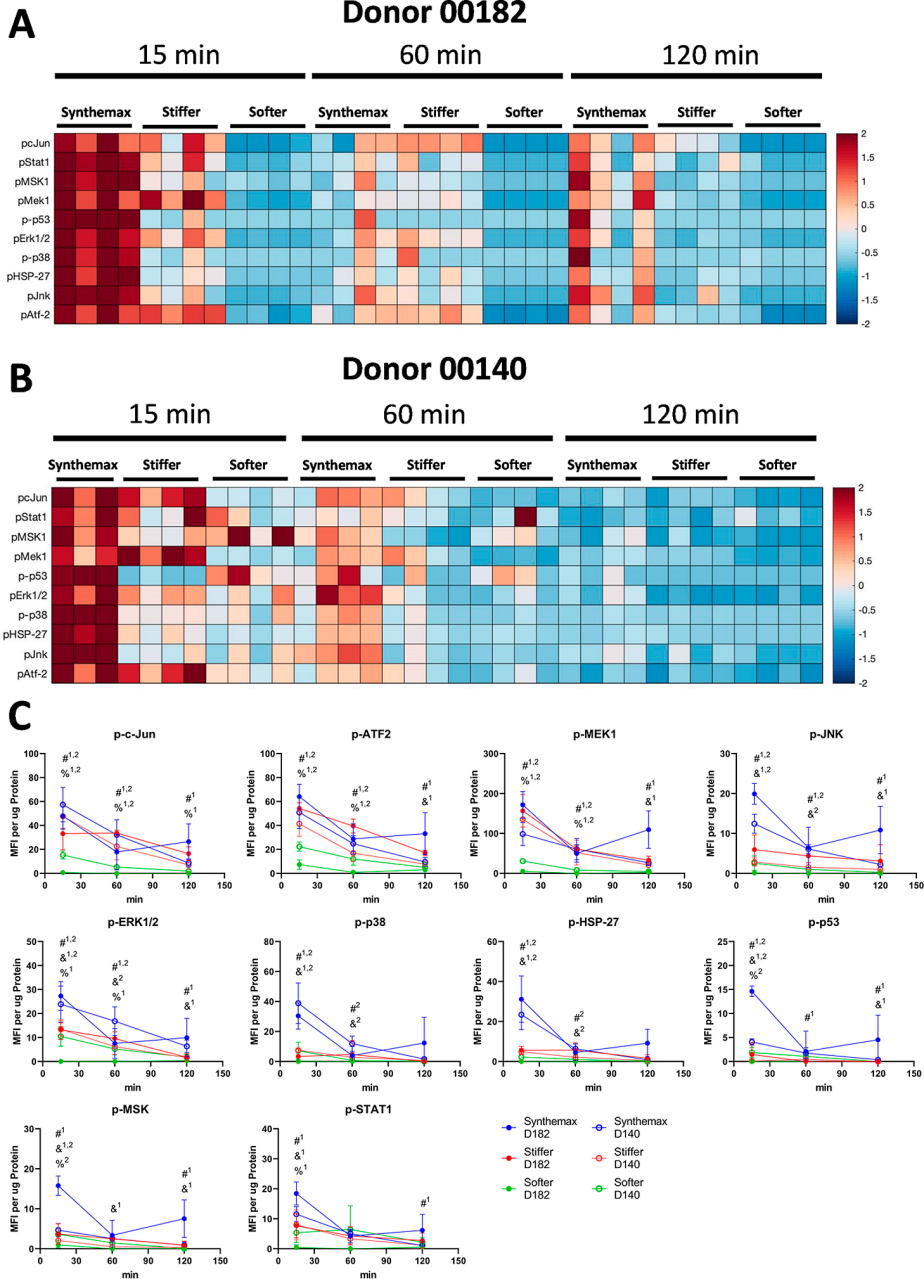
Stiffer and softer μCs reduce MAPK signaling in IL-1Ra-MSCs. Relative abundance of phosphorylated MAPK proteins in the lysate of IL-1Ra-MSCs from two donors. (A) Donor 00182 and (B) Donor 00140, respectively, cultured on different μCs, colors based on z-score. (C) Individual MAPK phosphoproteins differentially regulated between different μCs. &*p* < 0.05 vs Synthemax vs *stiffer*, #*p* < 0.05 Synthemax vs *softer*, %*p* < 0.05 *stiffer* vs *softer*. ^1^Observed in MSC Donor 00182, ^2^observed in MSC Donor 00140 (two-way ANOVA, Tukey *posthoc*).

All 10 quantified MAPK phosphoproteins in IL-1Ra-MSCs peaked 15 min after the start of agitation when cultured on all μC types for both cell donors (Figure 7A,B). 15 min after the start of agitation, differences in all 10 MAPK phosphoproteins were observed between IL-1Ra-MSCs cultured on different μCs using both cell donors. For both cell donors, a diminished increase in phosphorylated JNK, ERK1/2, p38, HSP-27, and p53 was observed when cells were cultured on stiffer and softer μCs compared to when cultured on Synthemax μCs (Figure 7C). Furthermore, in both donors, culture on softer μCs lowered the pc-Jun, pATF2, and pMEK1 content compared to culture on Synthemax and stiffer μCs (Figure 7C).

60 min after the start of agitation, differences in 5 MAPK phosphoproteins were observed between IL-1Ra-MSCs cultured on different μCs using both cell donors. Culture on softer μCs lowered pJNK and pERK1/2 content compared to culture on Synthemax μCs and lowered pc-Jun, pATF2, and pMEK1 content versus culture on both Synthemax and stiffer μCs (Figure 7C). 120 min after the start of agitation, no differences in MAPK phosphoprotein content were observed between IL-1Ra-MSCs cultured on different μCs types for either donor.

## 4 Discussion

In this study, two hydrogel formulations fabricated as μCs were used to improve secretion by unmodified MSCs and genetically modified MSCs. Culture on softer μCs increased expression and secretion of IL-1Ra by IL-1Ra-MSCs compared to culture on TCP-based Synthemax μCs, as well as improved MSC and IL-1Ra-MSC angiogenic activity. Additionally, culture on both stiffer and softer μCs differentially altered the secretion of multiple other immunomodulatory cytokines and chemokines versus culture on Synthemax μCs by both MSCs and IL-1Ra-MSCs. MAPK phosphoprotein analysis revealed that both cultures on stiffer and softer μCs reduce MAPK signaling in IL-1Ra-MSCs versus culture on Synthemax μCs, with particular phosphoproteins (pc-Jun, pATF2, and pMEK1) specifically downregulated on softer μCs. Taken together, these studies identify a potential strategy for further improving the secretory activity of genetically modified MSCs in a manner that may further improve their therapeutic activity and identify MAPK signaling proteins associated with such changes in secretory activity.

A fluidic device developed in our laboratory^27^ was used in these studies to generate hydrogel μCs for evaluation of MSC growth and secretome. These hydrogel formulations produced swelling ratios similar to those previously shown to alter MSC proliferation and secretory activity when used as a planar culture surface (30 and 100 kPa modulus).^7^ However, additional mechanical characterization is needed to fully determine the modulus following the μC fabrication. The fabrication device enabled control over hydrogel μC diameter (Figure 1), which was within the diameter range of the control Synthemax μCs (125–212 μm) at a throughput that was sufficient for performing small-scale experiments. Looking forward, other works using microfluidic devices utilizing water-in-oil emulsions to generate microparticles support feasibility of scaling-up fabrication methods to support μC MSC culture at scales more representative of clinical or commercial therapeutic cell production.^30,31^

Hydrogel μCs altered MSC proliferation in similar ways to that previously observed with the same formulations in planar hydrogel format.^7^ Softer μCs showed reduced MSC or IL-1Ra-MSC number at day 4 compared to stiffer or TCP-based Synthemax μCs (Figure 2). However, Synthemax and stiffer μCs failed to support MSC and IL-1Ra-MSC expansion at the same levels as that cultured on planar TCP surfaces. These observations were in accordance with the literature, where culturing MSCs in FBS-containing media formulations has shown limited or delayed growth kinetics when using a μC culture format due to slowed cell adhesion.^4,32^ Future studies using extended culture times or high-performance media may be needed to establish robust cell proliferation on these μCs.

In addition to impacting proliferation, the μC type also significantly impacted the secretory activity of both MSCs and genetically modified MSCs. As hypothesized, culture on softer μCs increased IL-1Ra secretion by IL-1Ra-MSCs compared to culture on Synthemax μCs (Figure 3A). This, to the best of our knowledge, is the first study demonstrating culture substrate-mediated amplification of transgene-encoded protein secretion. Amplification of transgene-encoded protein secretion is key to improving the functionality of genetically modified MSC-based therapies, which commonly suffer from low or transient gene expression.^15^ The use of biomaterials to promote secretion from genetically modified cells thus provides an alternative to more commonly used strategies, such as culture with soluble epigenetic modifiers^14^ or using tools for improving genomic integration.^33^

Culture on both hydrogel μCs also altered secretion of several endogenously expressed cytokines by both types of MSCs (secretion of endogenous factors was similar between MSCs and IL-Ra-MSCs on the same surface). MSCs and IL-1Ra-MSCs cultured on stiffer and softer μCs decreased secretion of several immunomodulatory factors, including IL-6, IL-8, GRO, and IP-10, versus those cultured on Synthemax μCs (Figure S3). Interestingly, some of the factors decreased when MSCs were cultured on stiffer and softer μCs were those associated with a pro-inflammatory, senescence-associated secretory phenotype, including IL-6, IL-8, GRO, and GM-CSF,^34^ suggesting that preculture on these μCs may reduce the development of inflammation once cells are implanted in vivo. Certain findings from the secretome analysis in this study were in contrast with previous results regarding the effects of planar 100 and 30 kPa culture surfaces, where MSCs on 30 kPa surfaces increased IL-6, IL-8, and GRO compared to those on TCP, and MSCs on 100 kPa surfaces did not alter IL-8 and GRO secretion compared to those on TCP.^7^ This discrepancy may be due to the impact of the proprietary vitronectin-derived coating on TCP-based Synthemax μCs. In addition, the μC format introduces substrate curvature and cell stretching, the latter of which has been shown to influence mechanosignaling pathways like yes-associated protein (YAP) signaling.^35^ While this work demonstrates the potential to further modulate the MSC secretome based on substrate shape and stiffness, additional study is required to better understand the components of μC culture contributing to differences in mechanosignaling and secretion between different culture formats.

Further, cells cultured on softer μCs showed altered secretion of multiple endogenous immunomodulatory factors versus the other μC types. MSCs and IL-1Ra-MSCs cultured on softer μCs significantly decreased the secretion of MCP-1, as well as increased the secretion of HGF and IL-10 (Figure S3). Decreased secretion of MCP-1 was consistent with the previous literature, where 30 kPa planar substrates were previously shown to reduce MCP-1 secretion in MSCs, albeit inconsistently.^7^ Similar to prior literature, culture on more compliant planar surfaces has also been shown to increase HGF secretion by MSCs.^36^ Such an increase in HGF secretion is most likely responsible for increases seen in this study in both MSC and IL-1Ra-MSC angiogenic activity from this μC type (Figure 5) since secretion of angiogenic factors HGF and VEGF was either enhanced or unchanged on softer μCs (Figure S3). Increased IL-10 secretion was unexpected, as it is not known to be endogenously secreted by MSCs. However, IL-10 can inhibit T cell proliferation and polarize macrophage toward a pro-regenerative phenotype in vitro,^37,38^ suggesting softer μCs may alter MSC immunomodulatory functionality. Further in vitro or in vivo assays to assess changes in MSC immunomodulatory activity on different μC formulations would be needed to examine the functional significance of improved IL-10 secretion.

Gene expression analysis of IL1RN, HGF, and IL-10 was performed to evaluate whether changes in gene transcription corresponded with the secretion of transgene-encoded and endogenous factors that were observed to have greater secretion on softer μCs. Cells on softer μCs increased IL1RN and HGF expression compared to both Synthemax and/or stiffer μCs as early as 1 day after seeding onto μCs (before starting agitation; Figure 6). While IL-10 gene expression was also evaluated, it was too low to be reliably quantified at any time point examined. These results suggest that culture on softer μCs upregulates expression of the transgene, and that may be the mechanism for the observed increase in secreted IL-1Ra.

In contrast to transgene expression, other endogenous genes encoding proteins with increased secretion were not all upregulated on softer μCs. Beyond increased gene expression, increased protein secretion may be due, in part, to enhanced intracellular transport of endogenous proteins, where stiffer culture substrates have been shown to increase trafficking of secreted proteins to the plasma membrane of hepatic stellate cells.^39^ Increased secretion may also be due to augmented exocytosis of proteins from the plasma membrane.^39,40^ Future work is needed to evaluate the relative contribution of transcriptional and post-translational cellular activity resulting in the amplified secretion of particular cytokines on softer μCs.

MAPK signaling of IL-1Ra-MSCs cultured on different μCs was evaluated to investigate signaling differences associated with proliferation, secretion, and angiogenic activity among conditions. This is the first study to evaluate MAPK signaling of MSCs cultured on μCs. MAPK signaling was chosen because it has been shown to be regulated by substrate stiffness in MSCs,^18,19^ as well as to regulate transgene expression in other cell types.^16^ MAPK signaling was evaluated 24 h after seeding, and immediately after the start of orbital agitation, to ensure adhesion of IL-1Ra-MSCs to μCs prior to the start of the experiment. These results thus captured the impact of agitation in combination with the μC type. Most differences observed between IL-1Ra-MSCs cultured on different μCs were observed 15 min after subjecting μCs to orbital shaking agitation, with MAPK phosphoprotein content, with fewer observed after 60 and 120 min (Figure 7). This finding was consistent with the previous literature showing that MAPK signaling changes induced from culture on planar substrates with different stiffnesses dissipated within 60 min of IL-1β stimulation.^41^ These data suggested early changes in MAPK signaling may cascade to result in changes in secretory activity of genetically modified MSCs on μCs over longer time periods, such as over 4 days. However, early changes in gene expression, such as the changes in IL-1Ra and HGF expression between μC types by day 1, cannot be explained by these results, as they were observed even before the start of agitation (Figure 6A,C). This indicates differential regulation of MAPK signaling identified in this study may not entirely correspond to cellular changes resulting in increased secretion on 30 kPa-formulation μCs. Therefore, further work to evaluate the role of MAPK signaling with and without μC agitation is needed to disentangle the impact of μC type from agitation.

In the context of cell number on the different μCs, comparing MAPK signaling may correspond to changes in MSC proliferation. IL-1Ra-MSCs cultured on softer μCs showed reduced phosphorylated c-Jun, ATF2, and MEK1 compared to Synthemax and stiffer μCs (Figure 7C). Decreased signaling of these factors is consistent with the previous literature showing reduced MSC proliferation is associated with lower c-Jun,^42^ ATF2,^43^ and MEK1 phosphorylation,^44^ something that was also observed in this study (Figure 2). However, other MAPK proteins associated with cell proliferation were not differentially regulated similarly to the previous literature. While ERK1/2 and p53 activity have been shown to increase and decrease MSC proliferation, respectively,^45,46^ neither corresponded with alterations in MSC proliferation in this study. These findings suggest that ERK1/2 and p53 signaling may not be entirely critical to supporting MSC proliferation on μCs during agitation.

## 5 Conclusions

In this study, we evaluated the changes in secretory activity in genetically modified MSCs cultured on different μC types. Two hydrogel formulations were fabricated as μCs with swelling ratios comparable to 100 and 30 kPa hydrogels, enabling the evaluation of substrate stiffness using highly scalable μC culture formats. Culturing MSCs and IL-1Ra-MSCs showed that softer μCs reduced cell numbers compared to Synthemax and stiffer μCs. IL-1Ra secretion by IL-1Ra-MSCs was increased when cultured on softer μCs, but several other cytokines and chemokines exhibited altered secretion when cultured on both stiffer and softer μCs compared with Synthemax μCs. Softer μCs also increased angiogenic activity of both MSCs and IL-1Ra-MSCs. Changes in secretory and angiogenic activity corresponded with an increased expression of IL-1Ra and HGF by IL-1Ra-MSCs cultured on softer μCs. Compared to the other substrates, downregulated phosphorylation of several proteins within the MAPK pathway, including c-Jun, ATF2, and MEK1, was observed when cells were cultured under agitation on softer μCs. As with any new culture technology, future studies using additional cell cycle analysis, a greater number of donors, longer culture time periods, and larger culture vessels (stirred-tank bioreactors) are needed to verify whether the effects of these different μCs are stable and ultimately applicable in clinical-scale culture formats. However, taken together, these novel findings suggest a strategy for improving the production of highly secretory genetically modified MSCs by using scalable culture formats, like μCs, together with identification of intracellular signaling pathways that may play a role in regulating the secretory and therapeutic activity of cells cultured on these carriers.

## Supporting Information Available

Additional experimental details, materials, methods, and results, including evaluation of AAV transduction methods and images from μC culture (PDF)
