# Supplementary material for: Poly(ethylene glycol)-Based Hydrogel Microcarriers Alter Secretory Activity of Genetically Modified Mesenchymal Stromal Cells
Source: ACS Biomater Sci Eng. 2023 Oct 31;9(11):6282–92. doi: 10.1021/acsbiomaterials.3c00954 (PMC10646834; doi:10.1021/acsbiomaterials.3c00954)
Supplement: Supplementary file 1 — ab3c00954_si_001.pdf [file ab3c00954_si_001.pdf]

Poly(ethylene glycol)-based hydrogel microcarriers alter secretory activity of genetically-modified mesenchymal stromal cells

Gilad Doron[1], Levi B. Wood[1,2,3], Robert E. Guldberg[4], Johnna S. Temenoff[1,3*]

[1]Wallace H. Coulter Department of Biomedical Engineering, Georgia Tech and Emory University

313 Ferst Dr. NW, Atlanta, GA 30332

[2]George W. Woodruff School of Mechanical Engineering, Georgia Institute of Technology

801 Ferst Dr. NW, Atlanta, GA 30318

[3]Parker H. Petit Institute for Bioengineering and Bioscience, Georgia Institute of Technology

315 Ferst Dr. NW, Atlanta, GA 30332

[4]Knight Campus for Accelerating Scientific Impact, University of Oregon

6231 University of Oregon, Eugene, OR 97403

*Corresponding author: johnna.temenoff@bme.gatech.edu

Supporting Methods

Material synthesis and functionalization

Poly(ethylene glycol) diacrylate (PEG-DA, 3.4 kDa) was synthesized according to previously published methods[1, 2]. Briefly, polyethylene glycol (PEG, 3.4kDa, Sigma) was reacted with 100% molar excess of acryolyl chloride in distilled dichloromethane, with triethylamine acting as a catalyst at a ratio of 1:1 with PEG. The reaction was allowed to continue overnight, after which the aqueous and organic phases were separated. The resulting PEG-DA was precipitated from organic phase in diethyl ether and vacuum dried.

Acryl-PEG-RGD was synthesized according to published methods[3, 4]. Integrin-engaging peptide GRGDS (Bachem) was dissolved in 50mM sodium bicarbonate buffer at pH 8.5. Acryl-PEG-succinimidyl valerate (Acrl-PEG-SVA, M_n ~ 3.4 kDa, Laysan Bio) was added at a molar ratio of 1:2 peptide:Acrl-PEG-SVA. After reacting for 3 hours with gentle stirring, the solution was dialyzed against H_2O using 3.5 kDa molecular weight cutoff tubing for 2 days and lyophilized. Acryl-PEG-GRGDS was purged under nitrogen gas and stored at -20 °C, protected from light prior to use.

MSC donor information supplied by manufacturer

Donor 00140: Lot 00140, Male, 26 years old, 9.4 population doublings upon receipt; Donor 00182: Lot 00182, Female, 25 years old, 8.9 population doublings upon receipt.

Supporting Results

Material fabrication

NMR imaging determined PEG acrylation yielded approximately 56% of PEG to have acrylate groups.

References

(1) Hahn, M. S.; Taite, L. J.; Moon, J. J.; Rowland, M. C.; Ruffino, K. A.; West, J. L. Photolithographic patterning of polyethylene glycol hydrogels. *Biomaterials* **2006**, *27* (12), 2519-2524. DOI: 10.1016/j.biomaterials.2005.11.045 From NLM.

(2) Krieger, J. R.; Ogle, M. E.; McFaline-Figueroa, J.; Segar, C. E.; Temenoff, J. S.; Botchwey, E. A. Spatially localized recruitment of anti-inflammatory monocytes by SDF-1alpha-releasing hydrogels enhances microvascular network remodeling. *Biomaterials* **2016**, *77*, 280-290. DOI: 10.1016/j.biomaterials.2015.10.045.

(3) Moon, J. J.; Saik, J. E.; Poché, R. A.; Leslie-Barbick, J. E.; Lee, S. H.; Smith, A. A.; Dickinson, M. E.; West, J. L. Biomimetic hydrogels with pro-angiogenic properties. *Biomaterials* **2010**, *31* (14), 3840-3847. DOI: 10.1016/j.biomaterials.2010.01.104 From NLM.

(4) Yang, P. J.; Levenston, M. E.; Temenoff, J. S. Modulation of mesenchymal stem cell shape in enzyme-sensitive hydrogels is decoupled from upregulation of fibroblast markers under cyclic tension. *Tissue Eng Part A* **2012**, *18* (21-22), 2365-2375. DOI: 10.1089/ten.TEA.2011.0727 From NLM.

Table S1. Immunomodulatory factors included for secretome characterization

EGF	IL-1β
Eotaxin	IL-4
FGF-2	IL-6
Flt-3L	IL-7
Fracktalkine	IL-8
G-CSF	IP-10
GM-CSF	MCP-1
GRO	MCP-3
HGF	PDGF-AA

IFNa2	RANTES
IFNy	SDF-1α
IL-10	TNFα
IL-1Ra	VEGF

Table S2. SYBR Green gene expression assays used to assess gene expression of IL-1Ra-MSCs on different μCs.

Target	SYBR Green Gene Expression Assay
IL1RN	qHsaCID0014491
HGF	qHsaCID0011441
IL10	qHsaCED0044704
B2M	qHsaCID0015347
GAPDH	qHsaCED0038674

Supporting figure legends

Figure S1. AAV transduction of MSCs yields cells that secrete IL-1Ra. A) Fluorescent imaging of MSCs (RoosterBio, lot #00140) 4 days after AAV transduction show expression of EGFP. **B)** MSCs transduced with AAVs showed significantly higher secretion of IL-1Ra as early as 2 days after transduction. * indicates 10^5MOI was higher than both 0 and 10^4 MOI (mean±SD, $p<0.05$, 2-way ANOVA, Tukey *post hoc*).

Figure S2. MSC and IL-1Ra-MSCs adhere to all μCs. Representative images of live MSCs from two donors cultured for 4 days on μCs, stained with Calcein AM (scale bar = 1000 um).

Figure S3. Individual immunomodulatory cytokines and chemokines whose secretion was altered by different μCs **in at least one donor.** Bars $p<0.05$, # $p<0.05$ vs MSC on same μC within same donor (2-way ANOVA, Tukey *post hoc*).
